# Can the Digital Economy Facilitate Carbon Emissions Decoupling? An Empirical Study Based on Provincial Data in China

**DOI:** 10.3390/ijerph19116800

**Published:** 2022-06-02

**Authors:** Kaiming Zhong, Hongyan Fu, Tinghui Li

**Affiliations:** 1 School of Economics and Statistics, Guangzhou University, Guangzhou 510006, China; 1964400076@e.gzhu.edu.cn; 2 School of Statistics and Mathematics, Central University of Finance and Economics, Beijing 100081, China

**Keywords:** digital economy, carbon emissions decoupling, PSTR model, social network analysis

## Abstract

The digital economy plays a dual role in the process of global carbon emissions decoupling; for this reason, its overall impact direction and mechanism are worth discussing. This paper attempts to answer the question of the role of the digital economy, based on a review of the existing literature. By constructing a panel smooth transition regression (PSTR) model, this paper empirically tests the effect of the digital economy on carbon emissions decoupling, based on panel data from 30 provinces in China from 2010 to 2019. In order to study the impact mechanism of the digital economy on carbon emissions decoupling, the mediating effect of industrial structure optimization is analyzed through a mediating effect model; the moderating effect is also explored by analyzing the network centrality characteristics of the digital economy. The core-periphery analysis method is adopted to group the samples to test the impact heterogeneity of the digital economy on carbon emissions decoupling. Based on this empirical analysis, the following conclusions are drawn. First, the digital economy has a promoting effect on carbon emissions decoupling, but this effect gradually weakens with the development of the digital economy. Second, the digital economy can promote carbon emissions decoupling through industrial structure optimization, and network centrality has a positive moderating effect on this mechanism. Third, heterogeneity exists in the promoting effect of the digital economy on carbon emissions decoupling, which is reflected in the different intensities of the promotion effect between the core nodes and the peripheral nodes in the network; the attenuation range of the promotion effect is also different when the regime switches.

## 1. Introduction

The digital economy has gradually become an increasingly important driving force for global economic growth [1]. Against this background, the relationship between the digital economy and carbon emissions decoupling has attracted much attention. On the one hand, the role of digital technology in reducing emissions has been widely recognized all over the world. In the report “Digitalization and Energy”, published by the International Energy Agency (IEA), it is predicted that through the large-scale use of digital technologies, in the European Union alone, increased storage and digitally-enabled demand response could reduce the curtailment of solar photovoltaics (PV) and wind power from 7 to 1.6% in 2040, avoiding 30 million tonnes of carbon dioxide emissions in 2040 [2]. On the other hand, the massive energy consumption brought about by the digital infrastructure has also raised concerns about carbon emissions. Research from the German Information Technology Association (ITG) in 2020 shows that the manufacturing and operation of digital equipment and infrastructure directly generate 1.8 to 3.2% of global greenhouse gas emissions [3]. In this context, it is of great practical significance to clarify the relationship between the digital economy and carbon emissions decoupling, which is conducive to grasping the positioning of the digital economy in the process of global carbon emissions decoupling.

In the existing studies, there is little direct research on the relationship between the digital economy and carbon emissions decoupling. The relevant research can be divided into two categories. The first is research on the evolution characteristics and influencing factors of carbon emissions decoupling, and the second is direct research into the role of the digital economy in terms of carbon emissions.

Some of the relevant literature on the evolution characteristics of carbon emissions decoupling analyzes the state of carbon emissions decoupling in a region by calculating the carbon emissions decoupling coefficient. For example, Mikayilov et al. studied the relationship between carbon dioxide emissions and GDP, based on the data of 12 Western European countries from 1861 to 2015. It was found that among the 12 European countries, 8 countries have a slower growth of emissions than of GDP and are in a relatively strong decoupling state [4]. Zhao, X. et al. studied the decoupling of China’s carbon dioxide emissions from industrial growth from 1993 to 2013 and found that China’s industrial sector was generally in a weak decoupling state during this period [5]. Xie, P. et al. calculated the Tapio Decoupling Index of carbon dioxide emissions for China’s power industry from 1985 to 2017. The results show that the decoupling state of carbon dioxide emissions in the power industry has mainly been characterized by expansionary negative decoupling and weak decoupling, and the current strong decoupling state has only existed for 5 years [6]. Other scholars have studied the factors affecting the decoupling coefficient using decomposition analysis. For example, Xu, S. et al. decomposed the carbon emissions decoupling coefficient of China’s fossil energy consumption through the logarithmic mean Divisia index, and they found that the economic output effect significantly enhanced the decoupling, while the energy intensity effect greatly reduced the decoupling, and the energy structure and the economic structure effects had a slight impact on the decoupling [7]. Chen, J. et al. also analyzed the impact of technical and non-technical factors on the carbon dioxide decoupling coefficient of OECD (Organization for Economic Co-operation and Development) countries by the logarithmic mean Divisia index. They believed that the impact of technical factors was greater than that of non-technical factors, and their impact directions were usually opposite [8]. Raza et al. decomposed the carbon emissions decoupling coefficient of Pakistan’s transport sector from 1984 to 2018 and found that the carbon dioxide coefficient effect is the factor that increases the decoupling coefficient, while the economic growth effect is the factor that decreases the decoupling coefficient [9].

In the literature that directly studies the role of the digital economy regarding carbon emissions, different scholars have put forward various views, which can be divided into the following three types. The first view is that the development of the digital economy will increase carbon emissions. For example, based on the panel data of Chinese provinces, Qiang, M. et al. found that the digital economy had a negative impact on carbon emission based on consumption, using the quantile regression method [10]. Avom et al. discovered that the use of information and communications technology (ICT) increased carbon dioxide emissions in 21 sub-Saharan African countries from 1996 to 2014 [11]. The second view is that the development of the digital economy can reduce carbon emissions. For example, based on OECD data, Wang, L. et al. discussed the impact and mechanism of digital technology innovation and spillover on China’s carbon emission intensity, and they suggested that digital technology could promote energy conservation and emission reduction [12]. Based on the panel data of 30 provinces in China from 2011 to 2017, Li, Y. et al. conducted research using the expanded STIRPAT (Stochastic Impacts by Regression on Population, Affluence and Technology) model and found that the digital economy can reduce carbon emissions via the moderating effect of the energy structure [13]. The third view holds that the relationship between the digital economy and carbon emissions is not simply linear but possesses certain nonlinear characteristics. For example, Li, X. et al. introduced the digital economy variable into the Solow growth model and conducted research based on the panel data of 190 countries from 2005 to 2016. They found that there is an inverted U-shaped, nonlinear relationship between carbon dioxide emissions and the digital economy [14]. Chien et al. studied the role of ICT in sustainable development in BRICS countries (Brazil, Russia, India, China and South Africa) from 1995 to 2018. The results showed that ICT significantly reduced carbon dioxide emissions only at a lower level of emissions [15].

As discussed above, the existing literature has tested the relationship between the digital economy and carbon emissions using different methods, which lays a foundation for subsequent research. However, at present, most of the literature directly studies the relationship between the digital economy and carbon emissions without considering the decoupling theory, which leaves a gap for the research presented in this paper. There are some limitations in using carbon emission indicators directly as explained variables for research because the result does not reflect the proper balance between economic development and the environment. Similar to the contribution that lean technologies can make to sustainable development, the technological changes brought about by the digital economy should also aim to achieve sustainable development [16,17]. Sustainable development theories emphasize promoting economic development without exceeding the carrying capacity of the earth, to ensure ecological sustainability [18]. The key of the sustainable development theory is how to reduce overall emissions while maintaining a high pace of economic development [4]. It can be seen that according to sustainability theory, reducing carbon emissions is not an isolated process. If reducing carbon emissions is at the cost of causing severe losses to economic growth, this emission reduction is not in line with the concept of sustainable development. Compared with the direct use of carbon emissions as the explained variable, the decoupling theory can more appropriately reflect the concept of sustainable development. Therefore, this paper attempts to introduce the concept of decoupling based on existing research, taking the decoupling coefficient of carbon emissions as the explained variable and digital economy as the explanatory variable, to study the relationship between them. Furthermore, the mediating effect is tested to explore the impact mechanism. The conclusions in the existing literature on the relationship between the two are quite diverse. One of the possible reasons is that the impact of the status of the digital economy has not been considered [19], because the impact of individual digital economy development on carbon emissions decoupling may vary due to its anomalous status. Therefore, this paper adopts a social network analysis method and introduces the network centrality variable to further analyze the possible impact of the digital economy’s status, from the perspective of the overall network.

The possible marginal contributions of this paper are as follows. First, the impact of the digital economy on carbon emissions decoupling is tested. This paper examines the impact of the digital economy on carbon emissions, based on the duality of the impact, making the relationship between them controversial. This research is conducive to further reflecting the transformation of the dependence of economic growth on carbon emissions and the role of the digital economy in this process. Second, network centrality is included in the analysis. The social network analysis method can help explore the status characteristics (i.e., network centrality) of individuals in the population by abstracting several individuals and their connections, to create a complex network of nodes and links. Incorporating the network centrality variable into the analysis framework is conducive to exploring the possible impacts of the network characteristics of the digital economy on carbon emissions decoupling.

The structure of the rest of this paper is as follows. Section 2 elaborates the research scheme; that is, based on the theoretical analysis of the impact of the digital economy on carbon emissions decoupling, the primary research hypotheses are put forward and the econometric models are set. Section 3 measures the variables, that is, measuring the variables in the models based on the data source explanation. Section 4 conducts econometric tests, wherein the relationship between the digital economy and carbon emissions decoupling is analyzed through benchmark regression and the appropriate robustness tests. Section 5 further discusses the transmission path of the digital economy affecting carbon emissions decoupling on the basis of econometric tests, as well as the possible impacts of the digital economy on network centrality. Section 6 summarizes our conclusions and puts forward the relevant policy implications.

## 2. Research Scheme

### 2.1. Theoretical Analysis and Research Hypothesis

Firstly, regarding the relationship between the digital economy and carbon emissions decoupling, it is difficult to give a simple answer to this question due to the complexity of the digital economy itself; the conclusions here will be different from the viewpoints of different angles of the digital economy. On the one hand, the core industries in the digital economy, such as the information technology industry, have a technological spillover effect that can promote industrial structural transformation through technological empowerment, decrease the proportion of the manufacturing industry, and increase the proportion of the service industry, thereby reducing carbon emissions [11,19]. From this point of view, the digital economy should promote carbon emissions decoupling. On the other hand, the core industries of the digital economy will also generate substantial carbon emissions, which will increase continuously with the development of the digital economy. From this perspective, there are also factors within the digital economy that inhibit the decoupling of carbon emissions. Under such factors, the relationship between the digital economy and carbon emissions decoupling may not represent a simple linear relationship. The environmental Kuznets curve (EKC) theory proposes that there is a nonlinear “inverted U-shaped” relationship between economic growth and environmental pressure; that is, with economic growth, the changing trend of environmental pressure first rises and then falls, and that there is an “inflection point” between the two [20,21,22]. Some studies have shown that this relationship can still be reflected after the environmental impact is embodied in carbon dioxide and other emissions [23]. However, it is still controversial whether EKC is established in terms of carbon emission. A typical criticism holds that the pollution caused by carbon dioxide emissions is cumulative and is difficult to eliminate with economic growth [24,25]. Nevertheless, the EKC hypothesis at least shows that there may be a staged difference in the effect of economic growth on carbon emissions. Therefore, this paper suggests that the digital economy, as a new economic form, may have a different effect on the decoupling of carbon emissions from the EKC in terms of the direction, but there is also the possibility of some staged differences; that is, with the continuous development of the digital economy, its role in carbon emissions decoupling will change. Based on this theory, the current paper proposes the following hypothesis:

**Hypothesis** **1** **(H1).** *The digital economy promotes carbon emissions decoupling, on the whole, but there is a nonlinear relationship between the two.*

Secondly, this paper analyzes the mechanism of the effect of the digital economy on carbon emissions decoupling from two perspectives. On the one hand, the digital economy can optimize the current industrial structure. Since the digital economy takes digital knowledge and information as being essential production factors, with digital technology as the core driving force [26], the development of the digital economy itself is accompanied by a digital transformation from resource- and labor-intensive industries to technology-intensive industries, thus optimizing the industrial structure. In addition, the digital industrialization brought by the digital economy has led to the birth of many emerging industries, most of which are in relatively low-emission, technology-intensive fields. The increasing proportion of such industries in the economic structure also promotes the optimization of the industrial structure. On the other hand, the optimization of this industrial structure can promote the decoupling of carbon emissions. In terms of the sources of carbon emissions, statistics from the International Energy Agency (IEA) show that in 2020, global carbon emissions mainly came from three fields: energy power generation and heating, transportation, and manufacturing and construction, accounting for 43%, 26%, and 11% of emissions, respectively [27]. It is clear that in these three industries, the leading producers of carbon emissions are the various industrial sectors in the secondary industry. In theory, the proportion of “dirty industries” in the broad industrial sector can be reduced gradually through industrial structure optimization [28]. Industrial structure optimization means that the proportion of the tertiary industry represented by technology-intensive industries increases, most of which are clean industries exhibiting high efficiency and low emissions. Their substitution for “dirty industries” is conducive to decoupling economic growth from carbon emissions. Based on the above analysis, this paper proposes the following hypothesis:

**Hypothesis** **2** **(H2).** *The digital economy can promote the decoupling of carbon emissions by optimizing the industrial structure.*

Thirdly, this paper analyzes the influence of network centrality on the digital economy, from the following two perspectives.

On the one hand, network centrality may affect the impact mechanism of the digital economy regarding carbon emissions decoupling. In the digital economy network, nodes have different network centralities because of their different positions in the overall network. Network centrality measures the digital economy status of a node. The higher the network centrality, the nearer the digital economy is to the core of the whole network. The core’s status in the network is likely to lead to the emergence of digital economy agglomeration. In his book *Principles of Economics,* Marshall mentioned that an important manifestation of economic agglomeration is the lock-in effect; that is, once an industry chooses a certain location, it tends to remain centered in that location for a long time [29]. The nodes at the core of the digital economy network are often those regions with a relatively developed economy, which are in an advantageous position in terms of the factor endowments related to the digital economy. Digital economy agglomeration promotes technology-intensive industries related to the digital economy, encouraging them to develop and expand continuously in a concentrated manner in the region, resulting in the optimization and upgrading of the industrial structure. Based on the above analysis, this paper proposes the following hypothesis:

**Hypothesis** **3** **(H3).** *Network centrality has a positive mediating effect on the process of the digital economy’s optimization of the industrial structure.*

Fourthly, network centrality may lead to the heterogeneity of the digital economy affecting carbon emissions decoupling. According to the core–peripheral theory, once a certain area forms a start-up industry, economic development will concentrate in the area near the starting point, due to the comprehensive action of various factors, making the area a core area; the peripheral area controlled by the core area is called the edge area [30,31]. There is a similar core–peripheral structure in the digital economy network, and network centrality is considered to be a criterion when quantitatively distinguishing the core and edge nodes. The location difference between the core and the edge areas in the digital economy network leads to industrial transfer between the two nodes, resulting in the heterogeneity of carbon emissions decoupling being promoted by the digital economy. The nodes in the core position in the digital economy network control the edge nodes. The core nodes can continuously obtain the production factors of the digital economy from the edge nodes and can transfer the replaced industries to the edge nodes after optimizing their industrial structure. As a result, when the digital economy develops, although the edge nodes can also enjoy the carbon emissions decoupling effect brought by the digital economy, their decoupling effect regarding carbon emissions will be more restrained and lower than that of the core nodes because the areas must accommodate the relocation and transfer of “dirty industries” from the core nodes. Based on this finding, this paper puts forward the following hypothesis:

**Hypothesis** **4** **(H4).** *The digital economy at the core of the network plays a stronger role in promoting carbon emissions decoupling.*

### 2.2. Model Setting

Based on H1, this paper adopts the PSTR model as the benchmark model, taking the carbon emissions decoupling coefficient as the explained variable and the digital economy as the core explanatory variable, to analyze the possible nonlinear relationship between the digital economy and carbon emissions decoupling. In 1999, Hansen proposed the well-known panel threshold model (PTR) [32] to study the nonlinear relationship between variables using panel data. The panel smooth transition regression (PSTR) model was developed on the basis of the PTR model. By introducing a smooth transition function, the PSTR model addresses the problem that the regression coefficients of the explanatory variable of the PTR model will jump on both sides of the threshold value, so the new model can achieve a smooth transition around the threshold [33]. In terms of the issues studied in this paper, if the role of the digital economy in decoupling carbon emissions undergoes a regional shift, this transition should be a gradual and smooth process rather than a sudden change. Therefore, the use of the PSTR model can more accurately reflect the possible regime transition process of the role of the digital economy on carbon emissions decoupling. The specific structure of the PSTR model set in this paper is shown in Formula (1):

(1) $$F_{{CO2}_{it}}=c+\beta_{0}DE_{it}+\beta X_{it}+\overset{r}{\underset{j=1}{\sum }}\beta_{j}DE_{it}G_{j}\left(q_{it};\gamma_{j},c_{j}\right)+u_{i}+\varepsilon_{it}$$

where $F_{{CO2}_{it}}$ represents the decoupling coefficient of carbon emissions of province i in year t; $DE_{it}$ stands for the digital economy development index of province i in year t; $X_{it}$ is a series of control variables; $u_{i}$ represents the individual fixed effect; $\varepsilon_{it}$ is the random error term. The coefficient $\beta_{0}$ represents the impact of the digital economy on carbon emissions decoupling, and this is the main parameter that this paper focuses on.

$G_{j}\left(q_{it};\gamma_{j},c_{j}\right)$ is the jth transition function, which is a continuous function with a value range of [0, 1]. Generally, the logistic function is adopted; that is:

(2) $$G_{j}\left(q_{it};\gamma_{j},c_{j}\right)=1/\left[1+exp\left(-\gamma_{j}\overset{m}{\underset{j=1}{\prod }}\left(q_{it}-c_{j}\right)\right)\right]$$

In Formula (2), $q_{it}$ is the transition variable. In order to study the impact of the digital economy on carbon emissions decoupling in different digital economy development levels, the transition variable set in this paper is the digital economy development level itself, so $q_{it}$ = $DE_{it}$. $\gamma_{j}$ is the smoothing parameter, which determines the smoothness of the transition. When $\gamma_{j}\to \infty$, the form of the model is close to the PTR model; when $\gamma_{j}\to 0$, the model changes into an ordinary fixed-effect model. $c_{j}$ is the threshold value, and the model will then have a smooth regime transition near $c_{j}$.

## 3. Variable Measurement

### 3.1. Measurement of Carbon Emissions Decoupling

Regarding the selection of the carbon emissions decoupling coefficients, there are two main decoupling coefficients used in the current mainstream research. One is the OECD decoupling coefficient proposed by the OECD [34], and the other is the Tapio decoupling coefficient proposed by Tapio in 2005 [35]. The calculation formulae of these two decoupling coefficients are as follows:

(3) F=1−EtiYtiEt0Yt0.

Formula (3) is the OECD decoupling coefficient, where *E* is the pollutant emissions; *Y* is the regional GDP; $t_{i}$ is the final year; $t_{0}$ is the base year.

(4) $$\varepsilon =\frac{E_{t+1}-E_{t}}{E_{t}}/\frac{Y_{t+1}-Y_{t}}{Y_{t}}$$

Formula (4) is the Tapio decoupling coefficient, where *E* and *Y* represent pollutant emissions and regional GDP; *t* + 1 is the current period and *t* is the previous period.

The Tapio decoupling coefficient measures decoupling by calculating the elasticity. Compared with the OECD decoupling coefficient, it has the advantage that there is no need to select the base period. However, for trend-based research, the Tapio decoupling coefficient has defects when exploring long-term relationships and it is highly sensitive to short-term policies [36]. Since decoupling should not be a sudden process but instead a stable and continuous separation of pollutant emissions and economic growth in the long term, it requires a certain time period and exacts an economic cost [37]. From the perspective of the trend research, the OECD decoupling coefficient is better than the Tapio decoupling coefficient. Therefore, this paper chooses the OECD decoupling coefficient to measure carbon emissions decoupling, to study the long-term trend change of carbon emissions decoupling under the influence of the digital economy. In this paper, 2009 is set as the base year of the measurement, and E in Formula (3) is concretely converted into carbon dioxide emissions. After calculation, the carbon emissions decoupling coefficient $F_{CO2}$ is obtained. The value range of $F_{CO2}$ is (−∞, 1); the larger the value, the greater the degree of carbon emissions decoupling.

### 3.2. Digital Economy Measurement

In terms of measuring the digital economy development level, referring to the research of Bei and Zhang [38], this paper comprehensively evaluates the development level of the digital economy from four different dimensions: the digital industry, digital innovation, digital users, and digital platform. After comprehensively considering the representativeness of indicators and the availability of relevant data, the final indicator system that is constructed is shown in Table 1.

For some missing data, this paper adopts a combination of the LOCF (last observation carried forward) method and the NOCB (next observation carried backward) method to supplement them.

In the selection of the comprehensive evaluation method, this paper selects the entropy method as the method of index weight calculation and comprehensive evaluation because the method has the advantage of objectivity. Unlike the subjective assignment method, the entropy method judges the weight of each index by the degree of discrete data, which avoids the arbitrariness and indiscipline caused by the subjective assignment process of the researcher. The specific steps to calculate the weight of each indicator and the comprehensive evaluation value of the digital economy using the entropy method are as follows.

First, we set $x_{ij}$ as the value of indicator j of evaluation object i, (i=1,2,⋯,m;j=1,2,⋯,n); the initial data matrix is as follows:

$$X=\left[\begin{array}{ccc} x_{11} & \cdots & x_{1n}\\ \vdots & \ddots & \vdots \\ x_{m1} & \cdots & x_{mn} \end{array}\right]$$

Second, all indicators are standardized to eliminate the impact of different dimensions on evaluation results, as shown in Formula (5):

(5) $$x_{ij}^{\prime }=\frac{x_{j}-x_{min}}{x_{max}-x_{min}};\;x_{ij}^{\prime }=\frac{x_{max}-x_{j}}{x_{max}-x_{min}}$$

In Formula (5), $x_{j}$ represents the value of indicator j; $x_{max}$ represents the maximum value of indicator j; $x_{min}$ represents the minimum value of indicator j; $x_{ij}$ represents the standardized value. If the indicator used is a positive one, the first equation in Formula (5) is used for processing; otherwise, the second equation is used for processing.

Third, to calculate the proportion of the ith evaluation value in indicator j, as shown in Formula (6):

(6) $$y_{ij}=\frac{x_{ij}^{\prime }}{\sum_{i=1}^{m}x_{ij}^{\prime }}\;\left(i=1,2\ldots ,m;j=1,2,\ldots ,n\right)$$

Fourth, to calculate the information entropy value and utility value of the jth indicator with Formulas (7) and (8):

(7) $$e_{j}=-K\overset{m}{\underset{i=1}{\sum }}y_{ij}lny_{ij}\;\left(K\;is\;a\;constant,K=\frac{1}{lnm}\right)$$

(8) $$d_{j}=1-e_{j}\;$$

Formula (7) is the calculation formula of the information entropy. The information utility value of an indicator is equal to the difference between 1 and the information entropy value, as shown in Formula (8). The information utility value directly affects the size of the weight. The bigger its value, the greater the indicator’s impact on the evaluation will be, and the greater the weight.

Fifth, to calculate the weight of each indicator, the proportion of the information utility value of the indicator among the information utility values of all indicators is used. The weight calculation formula of the jth indicator is shown in Formula (9):

(9) $$w_{j}=\frac{d_{j}}{\sum_{j=1}^{n}d_{j}}$$

Using the above methods, this paper calculates the weight of each indicator, as shown in Table 1.

Sixth, the comprehensive evaluation value of the evaluation object is calculated. The evaluation value of the evaluation object is calculated using the linear weighted summation formula, as shown in Formula (10):

(10) $$U=\overset{n}{\underset{j=1}{\sum }}y_{ij}w_{j}\;$$

Using the above six steps, this paper finally calculates the comprehensive evaluation value of the digital economy and uses it as an indicator to measure the development level of the digital economy.

From the results in Table 1, it can be seen that among all the three-level indicators in the indicator system, the proportion of urban unit employment in information transmission, computer services, and the software industry has the largest weight, accounting for 24.24%. The number of patents granted in the 5G industry has the lowest weight, which is 1.12%.

### 3.3. Measurement of the Network Centrality of the Digital Economy

In order to measure the network centrality of the digital economy, this paper first constructs a provincial digital economy network using the method of social network analysis, then calculates the network centrality variable of the digital economy. The specific process is as follows [39].

The first step is to use the improved gravity model to calculate the digital economic gravity among provinces, as shown in Formulas (11)–(13):

(11) $$GE_{m,n}=K_{m,n}\frac{\sqrt[{3}]{P_{m}G_{m}DE_{m}}\sqrt[{3}]{P_{n}G_{n}DE_{n}}}{ED_{m,n}^{2}}\;$$

(12) $$K_{m,n}=\frac{DE_{m}}{DE_{m}+DE_{n}}\;$$

(13) $$ED_{m,n}=\frac{D_{m,n}}{GP_{m}-GP_{n}}\;\;$$

In Formulas (11) and (12), $GE_{m,n}$ represents the province-to-province digital economy attraction; $P_{m}$ and $P_{n}$ are the resident population of province m and province n, respectively; $G_{m}$ and $G_{n}$ are the real GDPs of province m and province n, respectively; $DE_{m}$ and $DE_{n}$ represent the digital economy development scores of province m and province n, respectively. $ED_{m,n}$ stands for the economic distance between province m and province n; $K_{m,n}$ is the weight coefficient, which reflects the asymmetry of the digital economy gravity between two provinces. In Formula (13), $D_{m,n}$ is the geographical distance between province m and province n; $GP_{m}$ and $GP_{n}$ represent the per capita GDP of province m and province n, respectively. Through the calculation of the gravity of the digital economy between provinces, the digital economy gravity matrix ${\left(Y_{m,n}\right)}_{30\times 30}$ can finally be constructed.

In the second step, the gravity matrix is converted into a symmetric bipartite matrix using the threshold method. The mean values of the elements in each row of the digital economy gravity matrix are taken as the threshold values, which are compared with the gravity $Y_{m,n}$ between every two nodes in the matrix. If the gravity is greater than the threshold value, it means that there is a digital economy connection between nodes: therefore, let it be 1; otherwise, let it be 0.

The third step is to calculate the measures of the digital economy’s network centrality. This paper uses the degree of centrality and the closeness centrality as the core measures of the digital economy’s network centrality [40,41]. The degree of centrality measures the number of nodes directly connected with a node in the network, which is represented by the symbol DC. The more of such connections there are, the more the node is in the center of the network. The calculation formula of the degree of centrality is as follows:

(14) $$DC_{i}=\underset{j=1}{\sum }a_{ij}/\left(n-1\right)\;$$

In Formula (14), *n* is the total number of provinces; $a_{ij}$ indicates whether there is a digital economy connection between province i and province j. If $a_{ij}\;$= 1, it means that there is a digital economy connection between the two provinces; otherwise, if $a_{ij}\;$= 0, there is no digital economy connection between them.

The closeness centrality is the reciprocal of the sum of the distances between a node and other nodes in the network, and it is represented by the symbol CC. The closeness centrality can measure the ability of a node to be “independent from other nodes” in the network. The stronger this ability is, the more direct associations the node can establish with other nodes in the network, and the closer it is to the center of the network. The formula for calculating the closeness centrality is as follows:

(15) $$CC_{i}^{-1}=\sum_{j=1}d\left(i,j\right)/\left(n-1\right)$$

In Formula (15), d(i,j) represents the distance from province i to province j in the digital economy network.

### 3.4. Description of Other Variables and Data Sources

In addition to the above variables, other types of variables used in this empirical study can be described as follows.

The first type of variable is the control variable. Based on the existing research, this paper selects the variables that may have an impact on carbon emissions decoupling as the control variables [42,43,44,45,46,47,48]. Specifically, they are urban population density (UPD), which is obtained by dividing the population of cities under the jurisdiction of each province by the urban area, taking as the logarithm the green coverage of the built-up area (GCBA); that is, the ratio of green areas of cities under the jurisdiction of each province compared to the total area of built-up areas. The logarithm of per capita GDP (LGPC) is obtained by using the nominal per capita GDP of each province to de-inflate through the GDP index, with 2010 as the base period. Considering the theory of the environmental Kuznets curve, the square term of LGPC (STLGPC) is also taken as one of the control variables. Foreign direct investment (FDI) is represented by the amount of actually utilized foreign capital, creating the logarithm.

The second type of variable is the mediating variable. According to our theoretical analysis and the research hypotheses, this paper selects the industrial structure optimization as the mediating variable, which is represented by calculating the ratio of the output value of the tertiary industry to the output value of the secondary industry. All empirical analyses in this paper use the annual data for 30 Chinese provinces from 2010 to 2019, which can be considered as panel data (there are a total of 34 provincial administrative units in China, while the four provincial administrative regions of Hong Kong, Macau, Taiwan, and Tibet are excluded from our study, due to missing data). After examining the collected data, this paper ensures data integrity and consistency for all indicators.

The data of all indicators in this paper are obtained from public sources. Among them, the digital economy and digital economy network centrality are calculated by the authors, and the data required for the calculation are obtained from the China City Statistical Yearbook, China Statistical Yearbook, Enterprise Research Data—Digital Economy Industry Special Database, CEIC China Economic Database, China E-Commerce Yearbook, and China Internet Network Information Center. The carbon emissions decoupling index is also calculated by the author, and the data required for the calculation are obtained from the China Environment Statistical Yearbook. The data of the other variables are obtained from the China Statistical Yearbook and provincial statistical yearbooks.

After winsorizing 1% in each tail of all variables, the descriptive statistics of all variables in this paper are shown in Table 2.

From the descriptive statistics in Table 2, it can be seen that there is basically no singular value for each variable, which meets the basic empirical requirements. Specifically, for the carbon emissions decoupling coefficients, the average value is 0.371, and the maximum and minimum values are −0.063 and 0.666, respectively, indicating that within the time span selected in this paper, most provinces are in a relative decoupling state in terms of carbon emissions decoupling. There is still room for improvement in the intensity of decoupling.

**Figure 1:**
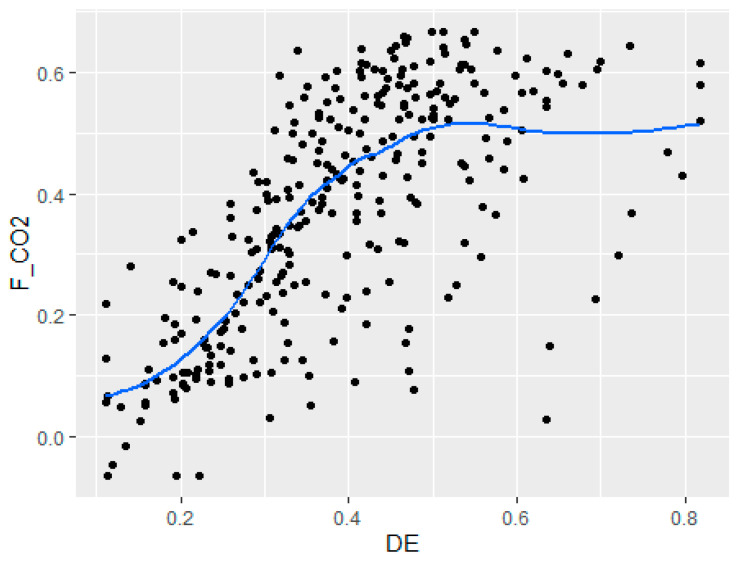
Digital economy and F_CO2_.

After obtaining all the data, this paper draws a scatter plot between the digital economy and carbon emissions decoupling, to initially verify the relationship between the two, as shown in Figure 1.

As can be seen from Figure 1, the relationship between the digital economy and carbon decoupling is positive overall, but there is a significant non-linear characteristic. This indicates that Hypothesis 1 of this paper is reasonable. In the next part of this paper, this nonlinear relationship will be empirically tested and quantitatively analyzed.

## 4. Econometric Tests of the Impact of the Digital Economy on Carbon Emissions Decoupling

### 4.1. The Impact of the Digital Economy on Carbon Emissions Decoupling

According to the previous model setting, the PSTR model is adopted in the benchmark regression to explore the impact of the digital economy on carbon emissions decoupling. Before using the PSTR model for parameter estimation, it is necessary to determine the transition function number *r* and the threshold value number *m* via testing, which can be divided into the following steps [49,50].

Firstly, a linear test is conducted to determine whether the model has at least one transition function; that is, the null hypothesis of the test meets γ=0. However, since the transition function of the PSTR model contains unknown parameters, the Taylor expansion is used to replace the transition function $G\left(q_{it};\gamma ,c\right)$. The results of the tests are shown in Table 3.

According to the results in Table 3, all *p*-values are less than 0.05 under the null hypothesis that the coefficient of the first-order to the fourth-order Taylor expansion is 0. Therefore, the null hypothesis is rejected; that is, γ≠0, and the model has at least one transition function.

Secondly, it is necessary to carry out the residual nonlinear test to see whether the nonlinear information extraction of the model is sufficient; that is, it is assumed that there is a second transition function $G_{2}\left(q_{it};\gamma_{2},c\right)$. The null hypothesis is $\gamma_{2}=0$, and the Taylor expansion is also used. The test results are shown in Table 4.

According to the results in Table 4, all *p*-values are greater than 0.05, so the null hypothesis is accepted; that is, $\gamma_{2}=0$, and the model has fully extracted the nonlinear information. Therefore, the model is optimal when there is only one transition function.

Finally, the Terasvirta sequential test is employed to test the optimal threshold number of the model. The results are shown in Table 5.

It can be seen from the results in Table 5 that the *p*-value of “b1 = 0|b2 = b3 = 0” is the smallest and the rejection of the null hypothesis is the strongest, so it can be considered that the optimal form of the model is established with only one threshold value.

Based on the above tests, it can be seen that the PSTR model in this paper is optimal when it has one transition function and one threshold value (that is, *r* = 1, *m* = 1). Therefore, the final form of the benchmark model is set as Formula (16) in this paper:

(16) $${F_{CO2}}_{it}=c+\beta_{0}DE_{it}+\beta X_{it}+\beta_{1}DE_{it}G\left(q_{it};\gamma ,c\right)+u_{i}+\varepsilon_{it}.$$

**Figure 2:**
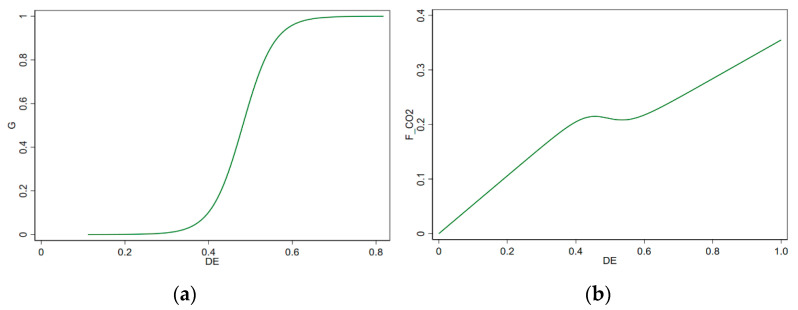
(**a**) The transition function; (**b**) Digital economy and decoupling coefficient.

After determining the numbers of the transition function and the threshold, this paper adopts the NLS method to estimate the parameters. The results of the benchmark regression are shown in Table 6. Figure 2a,b show the images of the transition function and the coefficient of the digital economy affecting carbon emissions decoupling, respectively.

It can be seen from the results in Table 6 that when the transition function $G\left(s_{it};c,\gamma \;\right)$ = 0, the model was in the low regime, and the regression coefficient of the digital economy was 0.530, which is significant at a significance level of 1%. Then, near the threshold DE = 0.482, the model underwent a smooth transition process. When the transition function $G\left(s_{it};c,\gamma \;\right)\;$= 1, the model shifted to a high regime. At this time, the regression coefficient of the digital economy decreased to 0.530 − 0.175 = 0.355, and it was still significant at the significance level of 1%. The benchmark regression results indicate that the digital economy (DE) has a significant promoting effect on carbon emissions decoupling ($F_{CO2}$) as a whole, but this promoting effect is different around the threshold DE = 0.482. When DE < 0.482, the promotion effect of the digital economy on carbon emissions decoupling is stronger than when DE > 0.482. This shows that the promotion effect of the digital economy on carbon emissions decoupling will be affected by the development level of the digital economy. When the development level of the regional digital economy is low, the process of carbon emissions decoupling can be effectively accelerated by developing the digital economy. However, when the digital economy develops to a certain extent, this promotion effect will gradually decline until it decays to a relatively low level and, then, remains stable. The results of the benchmark regression confirm H1 in this paper.

### 4.2. Robustness Tests

In order to ensure the reliability of the conclusions of this paper, this sub-section adopts three different methods to test the robustness of the benchmark regression results.

First of all, similar to carbon dioxide, sulfur dioxide is also a common emission in industrial production and in life and should be controlled. Although there are some differences in the harmfulness of carbon dioxide and sulfur dioxide, excessive emissions of sulfur dioxide also bring environmental problems. From this point of view, carbon dioxide and sulfur dioxide share similar properties. In addition, the two are coordinated in terms of emissions control. For example, China’s “Air Pollution Prevention and Control Law” stipulates that “coordinated control should be implemented on particulate matter, sulfur dioxide, nitrogen oxide, volatile organic compounds, ammonia, and other air pollutants and greenhouse gases”. Therefore, in the above benchmark model, the explained variable is replaced by the decoupling coefficient of sulfur dioxide emissions, which is calculated in a similar way to the decoupling coefficient of carbon emissions, and the parameters are estimated again under the same conditions.

Secondly, carbon emissions decoupling may also be affected by government policies [51]. Especially since the new leading group came into power in 2013, the government has paid more attention to environmental protection and emission reduction, promulgating a series of policies and laws related to environmental protection and emissions reduction to curb pollution, of which, limiting carbon emissions is the key measure. It is reasonable to believe that the emissions reduction policies issued after the 18th CPC National Congress will have an impact on carbon emissions decoupling. Therefore, referring to the practice of Shi et al. [52], this paper sets the policy dummy variable (POL) using 2013 as the turning point, acting as a control variable; that is, the variable value before 2013 is 0, and that after 2013 is 1. After adding the policy dummy variable into the original model, the parameters are estimated again, ceteris paribus.

Furthermore, in the process of constructing the digital economy indicator system and calculating the comprehensive evaluation value of digital economy development, some indicators will have missing data. Although this paper has used statistical methods to impute these missing data, they still have the potential to affect the benchmark regression results. In order to eliminate the possible impact of missing data, this paper eliminates all samples in the years with supplementary values and re-estimates the parameters.

The robustness test results using these three different methods are summarized in Table 7, where the three groups from left to right correspond to the parameter estimation results with the above three methods, respectively.

The results in Table 7 show that, under the three different robustness testing methods, although the numerical values of the regression coefficients of the digital economy have changed, their signs remain positive and their significance has not decreased significantly. In addition, the nonlinear relationship between the digital economy and carbon emissions decoupling has not changed. In the three sets of results, the model shows significant regime transition phenomena near the threshold value, and the regression coefficient on the low regime is higher than that on the high regime, which is the same as the nonlinear relationship between the two reflected in the benchmark regression. Therefore, it can be considered that the regression results of the benchmark model in this paper are robust.

### 4.3. Discussion on the Impact of the Digital Economy on Carbon Emissions Decoupling

This paper analyzes the regime transition phenomenon in the benchmark model, in terms of the respective evolutionary trends of carbon emissions and economic growth under the influence of the digital economy.

First, from the perspective of carbon emissions, there are stage differences in the role of the digital economy regarding carbon emissions. When the development of the digital economy is at a low level, industrial digitization integrates digital technology into the traditional production process, improves the production efficiency of traditional manufacturing enterprises, and improves the energy consumption control mode, thereby achieving a good effect in terms of emission reduction and pollution control. Meanwhile, digital industrialization in the digital economy promotes the upgrading of the industrial structure by giving birth to emerging industries, having a substitution effect on the secondary industry containing more high-emission industries. Therefore, at this stage, the development of the digital economy can significantly reduce carbon emissions. When digital economy development reaches a certain level, the continuous expansion of its scale may be restricted by some objective conditions, especially the constraints of the digital economy infrastructure. Therefore, after reaching this stage, continued digital economy development needs the support of larger-scale digital economy infrastructure construction and continuous operation, and this process will inevitably bring higher energy consumption and carbon dioxide emissions. Digital economy infrastructure, such as big data centers and cloud computing centers, will consume a great deal of electricity, which usually comes from coal-fired power plants, in terms of China’s energy structure, leading to significant carbon emissions. According to the 2019 statistics from the Greenpeace Organization and the North China Electric Power University, China’s data centers generated 99 million tons of carbon dioxide in 2018, equivalent to the annual emissions of about 21 million vehicles [53]. Therefore, as the scale of the digital economy continues to expand during this stage, its role in reducing emissions gradually declines. This process continues until the needs of infrastructure for the expansion of the digital economy are gradually met and a relative balance is reached between the two. After that, the emission reduction role of the digital economy still dominates, but since the digital economy infrastructure that has been built will still generate large carbon emissions, the emission reduction role of the digital economy will be suppressed to a certain extent at a relatively low institutional level at this time. In addition to the above reasons, the new pollution problems brought by the development of the digital economy are also one of the possible factors leading to the stage differences. With digital economy development, especially the development of digital industrialization, many emerging industries have been born and gradually expanded [54,55], the most typical example of which is the e-commerce industry. The emergence of e-commerce not only brings convenience to people’s lives but also brings new emission problems. For example, the large amount of waste packaging generated by online shopping need to be disposed of, as well as a large amount of fossil fuel energy being consumed by vehicles during commodity transportation, all of which have brought new carbon emission problems. As digital economy development reaches a certain level, the impact of these new pollution problems becomes more and more apparent. Therefore, the emission reduction role of the digital economy will be restrained to a certain extent.

Secondly, from the perspective of economic growth, unlike the effect on carbon emissions, the development of the digital economy makes a stable contribution to economic growth, as evidenced by many existing studies [56,57,58]. As an emerging economic form, the digital economy itself is part of the overall economy, so it is obvious that the development of the digital economy has a positive impact on economic growth. In addition, in terms of economic structure analysis, there is still much room to increase the share of the digital economy in China’s overall GDP. According to the “14th Five-Year Plan for Digital Economy Development” issued by the Chinese State Council, the value-added figures of China’s digital economy core industries account for 7.8% of the GDP in 2020, while according to the Plan, this share should reach 10% by 2025. This indicates that China’s digital economy will have great potential for a long time to come and can contribute to economic growth steadily without the phenomenon of regime transition.

From the above two aspects of the analysis, it is clear that digital economy development makes a stable contribution to economic growth, while there are stage differences in the contribution of the digital economy to carbon emissions. Influenced by these two aspects, the impact of the digital economy on carbon emissions decoupling shows a phenomenon of regime transition.

## 5. Further Analysis of the Digital Economy Affecting Carbon Emissions Decoupling

### 5.1. Analysis of the Impact Mechanism of the Digital Economy on Carbon Emissions Decoupling

The direct impact of the digital economy on carbon emissions decoupling has been analyzed above. However, in theory, the promotion of carbon emissions decoupling by the digital economy may not be a simple and direct process, but instead can only be realized through certain paths. Therefore, it is necessary to analyze the specific mechanism of the digital economy affecting carbon emissions decoupling. According to H2 and H3, this paper tests the mediating effect of industrial structure optimization and the moderating effect of network centrality, in order to explore the impact mechanism.

#### 5.1.1. Analysis on the Mediating Effect of Industrial Structure Optimization

Based on H2, this paper explores the possible mediating effect of industrial structure optimization on the process of the digital economy promoting carbon emissions decoupling by testing the regression coefficients step-by-step. The regression is divided into three stages [59]. The specific model settings are as in Formulas (17)–(19):

(17) $$\;F_{{CO2}_{it}}=c+\beta_{0}DE_{it}+\beta X_{it}+\beta_{1}DE_{it}G\left(q_{it};\gamma ,c\right)+u_{i}+\varepsilon_{it}$$

(18) $$LS_{it}=c^{\prime }+\beta_{0}^{\prime }DE_{it}+\beta^{\prime }X_{it}+u_{i}^{\prime }+\varepsilon_{it}^{\prime }$$

(19) $$F_{{CO2}_{it}}=c^{\prime \prime }+\beta_{0}^{\prime \prime }DE_{it}+\alpha LS_{it}+\beta^{\prime \prime }X_{it}+\beta_{1}^{\prime }DE_{it}G\left(q_{it};\gamma ,c\right)+u_{i}^{\prime \prime }+\varepsilon_{it}^{\prime \prime }$$

Among them, Formula (17) is the benchmark regression model, which has been verified earlier, so it will not be described repeatedly here. In Formulas (18) and (19), LS represents the mediating variable of industrial structure optimization, and the meanings of other variables are the same as those in the benchmark model. The parameter estimation results of Formulae (18) and (19) are shown in Table 8.

It can be seen from the empirical results of Formula (18) that the digital economy (DE) has a positive and significant effect on industrial structure optimization (LS) at the 1% significance level, indicating that the digital economy has a significant positive role in promoting industrial structure optimization. In Formula (19), the regression coefficient of the industrial structure optimization (LS) on carbon emissions decoupling ($F_{\mathrm{CO}2}$) is also positively significant at the 1% significance level, indicating that the industrial structure optimization also has a significant positive effect on carbon emissions decoupling. Based on the test results, it can be concluded that the digital economy has a positive impact on carbon emissions decoupling through industrial structure optimization, which plays a partial mediating role between the digital economy and carbon emissions decoupling. The results of the mediating effect test verified H2 of this paper.

The above test shows that the mechanism of the digital economy promoting carbon emissions decoupling is achieved through optimizing the industrial structure. This mechanism can be explained separately from the two core components of the digital economy. On the one hand, industrial digitization in the digital economy promotes the integration of digital technology and traditional industries through technological empowerment, and the tertiary industry is the fastest to integrate with digital technology. According to the statistics from the China Academy of Information and Communications Technology, in 2018, the digital economy accounted for 35.9%, 18.3%, and 7.3% of the added value of China’s service industry, industry, and agriculture, respectively, and the digitization degree of the service industry continues to lead the way [60]. This integration with digital technologies has brought about an increase in output and efficiency, which has greatly contributed to the scale growth of the service industry, thus driving the optimization of the industrial structure. On the other hand, the internal structure of digital industrialization in the digital economy is undergoing a continuous “softening” process. According to the data, in 2018, the software and information technology service industry and the Internet industry grew the fastest in terms of digital industrialization, with revenue increasing by 14.2% and 20.3% year-on-year, respectively [60]. This “softening” phenomenon in digital industrialization shows that various new business models centered on the service industry continue to develop and produce substitution effects on old industries, indicating a process of industrial structure optimization. Based on the above two aspects, it can be seen that these two core components of the digital economy can promote the optimization and upgrading of industrial structure, and the relatively low-emission tertiary industry has gradually increased in terms of its share of economic growth. Correspondingly, the role of high-emission enterprises in the secondary industry in terms of economic growth has been gradually replaced [61], so the decoupling of economic growth and carbon emissions has been significantly promoted under this mechanism.

#### 5.1.2. The Moderating Effect Analysis of Digital Economy Network Centrality

In order to further study whether the above-mentioned mechanism of the digital economy promoting carbon emissions decoupling is affected by digital economy network centrality, based on H3, this paper introduces network centrality variables and their respective interaction terms with the digital economy in Formula (18) to test the possible moderating effect of digital economy network centrality. The model set is shown in Formulas (20) and (21):

(20) $$LS_{it}=c+\alpha_{0}DE_{it}+\alpha_{1}inter_{1}+\alpha_{2}DC_{it}+\alpha X_{it}+u_{i}+\varepsilon_{it}^{\prime }$$

(21) $$LS_{it}=c^{\prime }+\alpha_{0}^{\prime }DE_{it}+\alpha_{1}^{\prime }inter_{2}+\alpha_{2}^{\prime }CC_{it}+\alpha^{\prime }X_{it}+u_{i}+\varepsilon_{it}^{\prime }$$

where DC and CC are the degree of centrality and the closeness centrality of the digital economy, respectively; $inter_{1}$ represents the interaction term between the degree of centrality and the digital economy; $inter_{2}$ stands for the interaction term between the closeness centrality and the digital economy. The meanings of other variables are the same as those in the previous models. The parameter estimation results for Formulas (20) and (21) are shown in Table 9.

In Table 9, Column (1) presents the moderating effect results of the degree of centrality, while Column (2) shows the moderating effect test results of the closeness centrality. It can be seen from the results that the regression coefficients of the interaction terms inter1 and inter2 are all significant at the 1% significance level, and the signs are all positive, indicating that both the degree of centrality (DC) and the closeness centrality (CC) of the digital economy network have significant positive moderating effects on the digital economy, promoting industrial structure optimization. The above empirical results verify H3 of this paper.

The above regression results show that the impact mechanism of the digital economy on carbon emissions decoupling is also related to the network centrality of the digital economy: the greater the degree and closeness centrality of the digital economy network, the stronger the impact mechanism of the digital economy on carbon emissions decoupling. It can be explained by the definition of the two kinds of network centrality.

Firstly, since the degree of centrality measures the number of other nodes directly connected to a node, in the digital economy network, the higher the degree of centrality of a node, the more nodes there are that have strong digital economy connections with it. This means that the node’s digital economy has a stronger ability to radiate outward, attracting more digital economy-related enterprises to engage in production activities at this node. According to the externality theory, this attraction produces a “snowball effect”; that is, more and more enterprises are willing to come together to share the benefits of the diversification and specialization of economic activities [62]. Most enterprises related to the digital economy are technology-intensive enterprises, so the snowballing agglomeration of these enterprises can directly promote the industrial structure optimization of the nodes, transforming the industrial structure from a resource-labor-intensive one to a technology-intensive one.

Secondly, since the closeness centrality measures the ability of a node to reach other nodes, in a digital economy network, the greater the closeness centrality of a node, the more convenient it is for it to trade with other nodes close to it, which means lower factor transportation costs. According to the theory of space economics, high transportation costs promote resource dispersion, while low transportation costs promote concentration. As a result, starting from any node in the network, the production factors of the digital economy tend to converge on the node with a large closeness centrality. Therefore, nodes with large closeness centrality in a network have strong resource concentration ability and can gather more production factors of the digital economy, which creates good conditions for the development of digital economy-related industries, thus improving the attractiveness of the nodes to digital economy-related enterprises and promoting industrial structure optimization.

**Figure 3:**
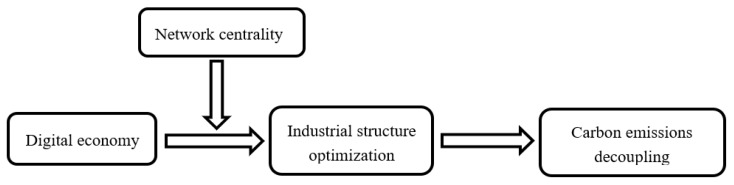
The mechanism of the digital economy affecting carbon emissions decoupling.

Based on the results of the above mechanism analysis, this paper holds that the mechanism by which the digital economy promotes carbon emissions decoupling has formed a mediating-effect model with moderating variables. The specific path is shown in Figure 3.

Figure 3 shows that the digital economy can promote carbon emissions decoupling by optimizing the industrial structure, and the network centrality of the digital economy has a positive moderating effect on this mechanism; specifically, it has a positive moderating effect on the digital economy, promoting industrial structure optimization.

### 5.2. Heterogeneity Analysis of the Digital Economy Affecting Carbon Emissions Decoupling

In order to further study whether there is heterogeneity in the promotion effect of the digital economy on carbon emissions decoupling in provinces with different network centrality, this paper uses the core-periphery method in social network analysis to categorize 30 sample provinces, sorting them into the core-province group and the peripheral province group. According to Borgatti, coreness can be regarded as a form of network centrality [63], so this grouping method can effectively test the heterogeneity caused by different network centralities. In the analysis results obtained in this paper, covering a total of ten years, the provinces that have been in a core position for more than 9 years are selected as the core group samples, and the remaining provinces are selected as the peripheral group samples. The grouping results are shown in Table 10.

According to the results in Table 10, the core group includes 9 provinces, while the peripheral group includes 21 provinces. The two groups of samples are regressed using the benchmark model, and the results are shown in Table 11.

In Table 11, Columns (1) and (2) are the regression results of the core group and the peripheral group, respectively. The empirical results show that the regression coefficients of the digital economy in the two groups are both positive and significant, and there is an obvious phenomenon of regime transition. Specifically, in the core group, when the transition function $G\left(s_{it};c,\gamma \;\right)$ = 0, the model is in a low regime, and the regression coefficient of the digital economy is 0.770; then, the model begins to transition smoothly around the threshold value DE = 0.435. When the transition function $G\left(s_{it};c,\gamma \;\right)$ = 1, the model transitions to the high regime, and the regression coefficient of the digital economy becomes 0.770 − 0.308 = 0.462. In the peripheral group, the threshold value is 0.531, indicating that the digital economy development level is higher than that of the core group when the regime transition occurs. In the low and high regimes, the regression coefficients of the peripheral group are 0.499 and 0.499 − 0.219 = 0.280, respectively, which are both smaller than those of the core group, indicating that the promotion effect of the digital economy on carbon emissions decoupling in the core group is significantly stronger than that in the peripheral group before and after the regime transition. This empirical result supports H4. In addition, in terms of the reduction range of the regression coefficient in the regime transition, the reduction range of the core group is 0.308, which is greater than that of the peripheral group, indicating that the effect of the digital economy on carbon emissions decoupling is attenuated more than that in the peripheral group when the regime transition took place.

Based on the above grouping regression results, it can be concluded that the promotion effect of the digital economy on carbon emissions decoupling is heterogeneous. There are differences in the promoting intensity between the core nodes and the peripheral nodes in the digital economy network, and the promoting intensity of the core group is always stronger than that of the peripheral group. This heterogeneity may be due to the fact that the agglomeration effect of the digital economy is more obvious at those nodes located at the core of the network. On the one hand, being affected by the agglomeration of production factors in the digital economy, the industries at the core of the network have a higher digital level, and the integration of digital technology and traditional production is deeper, while its role in the upgrading and transforming of high-energy-consuming enterprises is also more apparent. On the other hand, due to digital industrial agglomeration, the scale and development of the digital industrialization of the node at the core of the network are larger and faster. In addition, the polluting industries have begun to transfer to the peripheral nodes after they are replaced in the core node; therefore, the role of the digital economy in optimizing the industrial structure of the core node will be more obvious. Based on the above two reasons, the digital economy of provinces at the core of the network will play a stronger role in promoting carbon emissions decoupling.

In addition, from the nonlinear characteristics of the relationship between the digital economy and carbon emissions decoupling, it can be seen that the two groups have undergone a regime transition after the digital economy development reached a certain level, and the promotion effect gradually attenuated to a lower regime, but the attenuation range of the core group was greater than that of the peripheral group. The reason for the difference in the regime transition range between the two groups may be that the cities that are closer to the core of the network will bear greater carbon emissions, correspondingly, from the digital economy. In addition to reducing carbon emissions through various ways, the digital economy itself also brings new emissions. The continuous expansion and operation of the digital economy infrastructure, as well as the waste generated by emerging e-commerce platforms, have produced substantial carbon emissions. From the perspective of the network structure, cities in the center of the digital economy network have a digital economy agglomeration effect due to their close relationship with other nodes in the network. This is not only from the agglomeration of industries related to the digital economy but also the agglomeration of carbon emissions generated by them. Therefore, when the digital economy develops to a certain stage and its promoting effect on carbon emissions decoupling shifts to another regime, the core group will be influenced more strongly. Provinces at the core of the digital economy network tend to have more intensive distribution and a greater quantity of relevant infrastructures, so they consume more energy and produce more carbon emissions. Similarly, these provinces often experience more transactions on e-commerce platforms, so they generate more carbon emissions from waste packaging and transportation.

## 6. Conclusions and Policy Implications

This paper adopts the panel data of 30 provinces in China from 2010 to 2019 to investigate the impact of the digital economy on carbon emissions decoupling by constructing a PSTR model. On this basis, the specific mechanism of the digital economy affecting carbon emissions decoupling and the heterogeneity brought by different network centralities are explored. The following conclusions can be drawn.

First, the digital economy has a significant overall contribution to carbon emissions decoupling, and the conclusion still holds true after a series of robustness tests. This kind of promoting effect has nonlinear characteristics. When the digital economy develops to a certain extent, the promoting effect gradually decays until it remains stable at a low level.

Second, the digital economy promotes carbon emissions decoupling by optimizing the industrial structure, and the network centrality of the digital economy has a positive moderating effect on this mechanism. Specifically, it has a positive moderating effect on the process of the digital economy, promoting industrial structure optimization.

Third, heterogeneity exists in the role of the digital economy in promoting carbon emissions decoupling, which is caused by the different degrees of digital economy network centrality in various provinces. To be specific, in the network, the digital economy of the nodes at the core position has always been stronger than the nodes at the peripheral position in promoting carbon emissions decoupling, but the promotion effect of the core nodes is also attenuated more strongly when the regime transition occurs.

Based on the above conclusions, this paper draws the following policy implications. First, the government should encourage the innovative development of emerging digital industries, give full play to the role of the digital economy in optimizing the industrial structure, and promote the transformation and upgrading of resource- and labor-intensive industries to technology-intensive industries, to replace high-emission industrial sectors. Meanwhile, governments and enterprises should expand the application scope of digital technologies to more traditional production processes, to improve production efficiency and reduce emissions. Second, attention should be paid to the reasons for the attenuation of the promoting effect of the digital economy on carbon emissions decoupling; namely, the carbon emissions brought about by the digital economy’s development itself. For these new emission problems brought by the digital economy, the relevant regulations should be issued as soon as possible [64,65]. For example, relevant regulations should be issued for digital economy infrastructures, such as cloud computing centers and big data centers, to control carbon emissions, and green production and the use of clean energy should be encouraged [66,67]. Wastes from emerging industries, such as e-commerce, should be properly disposed of, and recycling should be encouraged. Furthermore, the network structure of the digital economy should be fully considered and utilized, and the emission reduction work of each node in the network should be planned as a whole. For nodes in the core positions in the network, on the one hand, the advantages of the agglomeration effect of the digital economy should be fully utilized to accelerate the development of industries related to the digital economy; on the other hand, the radiation role of the digital economy should be given full freedom for resources to be reasonably allocated and transferred to the peripheral nodes. For nodes at the periphery of the network, on the one hand, cross-regional digital trade should be encouraged, and digital economy connections should be established, with more nodes to improve their status in the network; on the other hand, the industrial transition of core nodes should be properly handled and then converted to retain their own developmental momentum, on the premise of controlling emissions.

Although this paper enriches the related research on the relationship between the digital economy and carbon emissions decoupling, there is still room for improvement. Firstly, the research in this paper is carried out within the spatial dimension of provinces, so whether the conclusions of this paper will change when applied to other spatial dimensions remains to be tested. Follow-up research can further test the impact of the digital economy on the decoupling in specific spatial dimensions, such as cities and countries. Secondly, due to the data availability, the time span of the data used in this paper is only 10 years; it remains to be tested whether the impact of the digital economy on carbon emissions decoupling will still be the same for a new regime over a longer time span. Furthermore, although the PSTR model is adopted in this paper to reasonably test the nonlinear characteristics of the digital economy affecting carbon emissions decoupling, there may still be a better model to better fit the relationship between them, but this depends on the development of relevant research on the panel data nonlinear model in follow-up studies.

## Figures and Tables

**Table 1:** Digital economy indicator system.

Level 1 Indicators	Level 2 Indicators	Level 3 Indicators	Weight
Level of digital economy development	Digital industry	Proportion of employment in urban units in information transmission, computer services, and software industries	24.24%
		Software business revenue (log)	3.47%
		The proportion of information transmission, computer services, and the software industry in the fixed assets of the whole society	11.41%
	Digital innovation	Number of patents granted for 5G industry (log)	1.12%
		Number of industrial Internet patents granted (log)	3.17%
		Number of e-commerce patent granted (log)	14.61%
	Digital user	Popularization rate of mobile telephones	6.88%
		Total amount of telecommunication services (log)	5.45%
		Number of Internet broadband access users per capita	11.23%
		Digital financial inclusion development (log)	5.72%
	Digital platform	Number of domain names (log)	7.18%
		Number of web pages (log)	1.53%
		Number of Internet users (log)	4.00%

**Table 2:** Descriptive statistics.

Item	Mean	sd	Min	Max
F_CO2_	0.371	0.189	−0.0630	0.666
DE	0.391	0.145	0.112	0.818
UPD	7.877	0.412	6.952	8.614
GCBA	0.392	0.0367	0.295	0.484
LGPC	10.70	0.462	9.482	11.78
STLGPC	114.7	9.937	89.90	138.8
FDI	12.80	1.651	7.310	15.09
LS	1.264	0.702	0.527	5.234
DC	0.250	0.0647	0.0690	0.414
CC	0.334	0.0487	0.213	0.547

**Table 3:** Linear tests.

H_0_	chi2	df1	df2	Prob
b1 = 0	17.6282	2	263	6.541 × 10^−8^
b1 = b2 = 0	16.3572	3	262	8.967 × 10^−10^
b1 = b2 = b3 = 0	14.0167	4	261	2.254 × 10^−10^
b1 = b2 = b3 = b4 = 0	11.5404	5	260	4.450 × 10^−10^

**Table 4:** Residual nonlinear test.

H_0_	chi2	df1	df2	Prob
b1 = 0	0.5738	1	263	0.4494
b1 = b2 = 0	0.5335	2	262	0.5872
b1 = b2 = b3 = 0	0.3638	3	261	0.7792
b1 = b2 = b3 = b4 = 0	0.3012	4	260	0.877

**Table 5:** Terasvirta sequential test.

H_0_	chi2	df1	df2	Prob
b1 = 0|b2 = b3 = 0	17.6282	2	263	6.541 × 10^−8^
b2 = 0|b3 = 0	11.9775	2	262	0.00001053
b3 = 0	9.2935	2	261	0.0001262

**Table 6:** Benchmark regression.

Item	F_CO2_	
	Linear (1)	Non-Linear (2)
DE	0.530 ***	−0.175 ***
	(6.540)	(−4.120)
UPD	−0.003	
	(−0.220)	
GCBA	0.206	
	(1.150)	
LGPC	−1.578 ***	
	(−5.040)	
STLGPC	0.106 ***	
	(7.120)	
FDI	0.008	
	(1.580)	
threshold1		0.482 ***
		(32.710)
Lngamma		3.277 ***
		(9.970)
Constant	4.777 ***	
	(100.750)	
R2	0.968	
Observations	300	

Robust standard errors in parentheses. *** *p* < 0.01.

**Table 7:** Robustness test.

Item	F_SO2_		F_CO2_		F_CO2_	
	Linear (1)	Non-Linear (2)	Linear (3)	Non-Linear (4)	Linear (5)	Non-Linear (6)
DE	0.370 ***	−0.150 **	0.496 ***	−0.145 ***	0.324 ***	−0.185 ***
	(3.040)	(−2.260)	(6.380)	(−3.780)	(3.410)	(−3.190)
UPD	−0.061 ***		−0.005		−0.022	
	(−3.600)		(−0.310)		(−1.190)	
GCBA	0.074		0.262		0.190	
	(0.370)		(1.470)		(0.890)	
LGPC	−2.648 ***		−1.529 ***		−0.982 *	
	(−8.000)		(−4.990)		(−1.910)	
STLGPC	0.162 ***		0.102 ***		0.087 ***	
	(10.320)		(6.960)		(3.590)	
FDI	0.004		0.007		0.005	
	(0.780)		(1.470)		(0.790)	
POL			0.021 **			
			(2.540)			
threshold1		0.432 ***		0.489 ***		0.470 ***
		(10.890)		(31.450)		(27.910)
Lngamma		3.072 ***		3.403 ***		3.258 ***
		(6.910)		(9.300)		(8.600)
Constant	10.480 ***		4.717 ***		0.852 ***	
	(91.690)		(101.870)		(112.980)	
R2	0.964		0.968		0.960	
Observations	300		300		180	

Robust standard errors in parentheses *** *p* < 0.01, ** *p* < 0.05, * *p* < 0.1.

**Table 8:** Mediating effect test results.

Item	LS	F_CO2_	
	(1)	Linear (2)	Non-Linear (3)
DE	1.718 ***	0.411 ***	−0.204 ***
	(4.240)	(4.760)	(−3.31)
LS		0.085 ***	
		(5.840)	
UPD	−0.227	0.015	
	(−1.630)	(0.990)	
GCBA	−0.849	0.283 *	
	(−0.750)	(1.670)	
LGPC	−6.059 *	−1.146 ***	
	(−1.760)	(−3.550)	
STLGPC	0.293 *	0.085 ***	
	(1.770)	(5.520)	
FDI	0.029	0.006	
	(0.690)	(1.200)	
threshold1			0.481 ***
			(31.550)
lngamma			3.069 ***
			(7.630)
Constant	33.538 *	2.358 ***	
	(1.810)	(116.550)	
R2	0.704	0.971	
Observations	300	300	300

Robust standard errors are in parentheses *** *p* < 0.01, * *p* < 0.1.

**Table 9:** Moderating effect test results of digital economy network centrality.

Item	LS	
	(1)	(2)
DE	0.688	−0.976
	(1.490)	(−1.27)
DC	−1.608 ***	
	(−4.370)	
inter1	3.766 ***	
	(4.370)	
CC		−2.945 ***
		(−3.05)
inter2		7.132 ***
		(3.80)
UPD	−0.201	−0.174
	(−1.480)	(−1.32)
GCBA	−0.946	−0.926
	(0.880)	(−0.88)
LGPC	−5.596	−4.371
	(−1.700)	(−1.35)
STLGPC	0.275 *	0.222
	(1.730)	(1.42)
FDI	0.021	0.004
	(0.570)	(0.13)
Constant	31.003 *	24.695
	(1.750)	(1.42)
R2	0.723	0.740
Observations	300	300

Robust standard errors in parentheses *** *p* < 0.01, * *p* < 0.1.

**Table 10:** Grouping results of the core-periphery analysis.

Core Group	Peripheral Group
Beijing, Tianjin, Shanghai, Jiangsu, Zhejiang, Shandong, Henan, Guangdong, and Gansu	Hebei, Shanxi, Inner Mongolia, Liaoning, Jilin, Heilongjiang, Fujian, Hunan, Hubei, Jiangxi, Guangxi, Hainan, Chongqing, Sichuan, Guizhou, Yunnan, Shaanxi, Gansu, Qinghai, Ningxia, and Xinjiang

**Table 11:** Heterogeneity test results.

Item	F_CO2_			
	The Core Group		The Peripheral Group	
	Linear (1)	Non-Linear (2)	Linear (1)	Non-Linear (2)
DE	0.770 ***	−0.308 **	0.499 ***	−0.219 **
	(3.260)	(−2.040)	(5.750)	(−2.310)
UPD	0.054		0.001	
	(1.510)		(0.030)	
GCBA	1.079 ***		0.110	
	(2.930)		(0.540)	
LGPC	−1.660 ***		−1.095 **	
	(−3.710)		(−2.330)	
STLGPC	0.107 ***		0.082 ***	
	(5.150)		(3.640)	
FDI	0.039 ***		0.002	
	(2.980)		(0.370)	
threshold1		0.435 ***		0.531 ***
		(16.830)		(17.630)
lngamma		2.893 ***		3.330 ***
		(6.120)		(5.850)
Constant	3.975 ***		2.492 ***	
	(65.990)		(88.670)	
R2	0.978		0.967	
Observations	90	90	210	210

Robust standard errors in parentheses. *** *p* < 0.01, ** *p* < 0.05.

## Data Availability

The data shown in this research are available on request.
